# Ketosis-Prone Type 2 Diabetes (Flatbush Diabetes) in Remission: A Report of Two Cases

**DOI:** 10.7759/cureus.28514

**Published:** 2022-08-28

**Authors:** Beisi Ji, SumathaChannapatna Suresh, Klynt Bally, Kamrun Naher, Mary A Banerji

**Affiliations:** 1 Internal Medicine, State University of New York Downstate Medical Center, New York, USA; 2 Endocrinology, State University of New York Downstate Medical Center, New York, USA

**Keywords:** subgroup a-β+, ketosis-prone type 2 diabetes, flatbush diabetes, remission, recovery of beta-cell function

## Abstract

Diabetic ketoacidosis (DKA) is a triad of uncontrolled hyperglycemia, metabolic acidosis, and increased total body ketone concentration. It is a well-known manifestation of type 1 diabetes mellitus (T1DM). However, it can also be the first presentation of type 2 diabetes mellitus (T2DM). This subtype of diabetes shares the characteristics of both T1DM and T2DM and is called 'Flatbush diabetes,' also known as 'ketosis-prone T2DM.' This article highlights the importance of early identification of ketosis-prone T2DM (KPD). We describe two cases of African American men with no past medical histories who presented with unproved DKA as the first presentation of T2DM requiring initial intensive insulin therapy, which was gradually withdrawn with the addition of metformin. Both patients are currently insulin independent for more than 15 months. It is an essential clinical entity to recognize as insulin independence positively impacts the quality of life and decreases the economic burden on the health care system.

## Introduction

It has been recognized since the 1980s that for some patients hospitalized with diabetic ketoacidosis (DKA), their clinical courses were more like those with type 2 diabetes mellitus (T2DM) [1]. Patients with ketosis-prone type 2 diabetes usually present with new-onset severe hyperglycemia and ketosis or diabetic ketoacidosis. The treatment is the same as diabetic ketoacidosis, which requires insulin, intravenous fluid, and electrolyte replacement. After being treated with insulin for several weeks to a few months, a large percentage of these patients could discontinue insulin injection, and their DM can be well-controlled with only lifestyle modification or lifestyle plus oral medications for many years. A small percentage of people still need insulin therapy. Evidence also shows that sulfonylurea therapy can prolong remissions in patients with KPD [2-4]. We, therefore, report two typical cases of ketosis-prone diabetes.

This article was previously presented as a meeting abstract at the Endocrine Society's annual meeting (ENDO) 2022 on 06/12/2022.

## Case presentation

Case 1

A 65-year-old African American man presented to the emergency room experiencing polydipsia, polyphagia, and polyuria for two weeks. These were the results of his examination on presentation: initial fingerstick glucose (FS) >500mg/dL, beta-hydroxybutyrate (BHB) of 4.32mmol/L, urine ketone positive, PH of 7.305, bicarbonate at 18mEq/L, anion gap (AG) of 29mEq/L, serum osmolality of 368mOsm/Kg, glucose of 1262mg/dL, hemoglobin A1c (HbA1C) of 12.9%, and body mass index (BMI) of 26.36kg/m2. The DKA mixed with hyperosmolar hyperglycemic syndrome (HHS), and new-onset T2DM was diagnosed. Precipitating factors such as infections, Covid-19, and myocardial infarction were ruled out. The patient was admitted to the medical intensive care unit (MICU) and received an insulin drip and fluid. He recovered uneventfully and was discharged on aspart 8u three times a day (TID) and glargine 20u every night at bedtime (QHS), and followed closely in our endocrinology clinic.

At one month of follow-up, fasting glucose reading from the log book was around 100mg/dL, pre-meal glucose ranged between 90-120mg/dL, glargine was decreased to 17u QHS, aspart was decreased to 7u TID, and metformin 1000 mg twice a day (BID) was started. At the three-month follow-up, HbA1C improved significantly to 6.7% without significant hypoglycemic episodes. The insulin dose was further reduced, and metformin 1000mg BID was continued. The B-cell autoantibodies were negative, and the C-peptide was 2.6nmol/L. The HbA1C was improved to 5.8% on a subsequent visit in the same year. Therefore, insulin was gradually withdrawn, and the metformin dose was decreased with close monitoring. The patient's hemoglobin level and renal function were within normal limits in all clinical follow-ups. Current medications include metformin 500mg BID and atorvastatin 40mg, with the most recent HbA1C at 6.0%. The patient’s current BMI is 27.41 kg/m2. He has been in remission for 18 months (Table 1).

**Table 1:** Timeline, HbA1C, and diabetic regimen HbA1C: Hemoglobin A1C, BID: Twice a day, TID: Three times a day, QHS: Every night at bedtime

Time	HbA1C	Diabetic regimen
Diagnosis	12.9%	Insulin drip, discharged on aspart 8u TID and glargine 20u QHS
One-month follow-up		Glargine 17u QHS, aspart 6u TID, metformin 1000mg BID
Three months follow-up	6.7%	Glargine 12u QHS, aspart 3u TID, metformin 1000mg BID
Six months follow-up	5.8%	Glargine 10u QHS, metformin 500mg BID
Nine months follow-up	5.5%	Metformin 500mg BID
One-year follow-up	5.7%	Metformin 500mg BID
Two-year follow-up	5.8%	Metformin 500mg BID
18 months follow-up	6.0%	Metformin 500mg BID

Case 2

A 24-year-old obese African American man with a family history of diabetes (two maternal aunts) presented to the emergency room with fatigue, polyuria, and polydipsia for the past three days. On arrival, these were his results: glucose of 1299mg/dL, BHB of 6.45mmol/L, urine ketone>80, PH at 7.248, bicarbonate of 15mEq/L, AG of 33mEq/L, HbA1C at 11.1%, and BMI of 37.7kg/m2. He was diagnosed with unprovoked DKA and new-onset DM. The patient recovered uneventfully and was discharged with aspart 6 units TID and glargine 25 units QHS.

He was seen two months later, and his fasting glucose was around 70mg/dL, so aspart was discontinued, glargine was decreased to 18 units QHS, and metformin 500mg BID was started. At the three-month follow-up, HbA1C dropped to 6.2% with medications and lifestyle changes. The patient reported only a few hypoglycemic episodes. Glargine was gradually decreased and stopped within a year of his diagnosis. Islet cell antibodies and GAD antibodies were negative. The C-peptide level was 6nmol/L. The patient’s current BMI is 37.57kg/m2. His current medications are metformin 1000mg BID and atorvastatin 10mg daily. His most recent HbA1C was 5.5%. He has been in remission for 15 months (Table 2).

**Table 2:** Timeline, HbA1C, and diabetic regimen HbA1C: Hemoglobin A1C, BID: Twice a day, TID: Three times a day, QHS: Every night at bedtime

Time	HbA1C	Diabetic regimen
Diagnosis	11.1%	Insulin drip, discharged on aspart 6u TID and glargine 25u QHS
Two months follow-up		Glargine 18u QHS, metformin 500mg BID
Three months follow-up	6.2%	Glargine 8u QHS, metformin 500mg BID
One-year follow-up	5.7%	Metformin 1000mg BID
15th-month follow-up	5.5%	Metformin 1000mg BID

## Discussion

Ketosis-prone diabetes is more commonly seen in middle-aged overweight or obese men with positive family history, metabolic syndrome, and people of African, Caribbean, Asian, or Hispanic descent [5-9]. These patients usually present with typical diabetic symptoms, i.e., polyuria, polydipsia, and weight loss for less than four to six weeks without any precipitating factors [9].

There are no gold standard tests to diagnose KPD. For all patients who are suspected of having KPD, their islet cell antibodies and C-peptide levels should be obtained to evaluate β cell autoimmunity and β cell secretory reserve. The KPD can be further divided into four subgroups according to the presence or absence of GAD 65, GAD 67 or islet antigen 2 (IA-2) autoantibodies (A+ or A-), and β-cell functional reserve (β+ or β-). The course of disease progression mainly depends on their β-cell functional reserve. Although A+β− and A−β− patients are genetically different, they both lack β-cell function. Their clinical course is similar to T1DM, and they require lifelong insulin therapy. For A-β+ and A+β+ patients, because they have a normal β-cell function, their clinical course is like those with T2DM, insulin dose can be slowly reduced, even discontinued, and an oral agent can be started if patients’ glucose remains at the goal [10,11].

Among KPD, the A−β+ group is the largest; nearly half of these patients present to the hospital with new-onset diabetes and develop DKA without precipitating factors (unprovoked A−β+ KPD), whereas the remainder has a history of diabetes and develop ketoacidosis due to stress, acute illness, or medication noncompliance (provoked A−β+ KPD) [12]. Patients with unprovoked A−β+ KPD have more likelihood of becoming insulin-independent and have better glycemic control than the latter [10,12].

Since many patients with KPD present with ketoacidosis, there is a high probability of it being misdiagnosed as T1DM. If that happens, these patients will be unnecessarily treated with long-term insulin as physicians will be under the impression that they need lifelong insulin for T1DM. Therefore, they are at an increased chance of developing side effects of insulin treatment i.e., hypoglycemia and weight gain. Long-term insulin injection and frequent self-monitoring can also decrease their quality of life, causing career limitations and economic burdens to patients and the healthcare system [8]. So it is extremely important to check β-cell autoantibodies and C-peptide levels one to three weeks after discharge at the first outpatient visit [13]. In our two cases, the first patient is a middle-aged male, and the second patient is an obese young adult who presented with new-onset diabetes with unprovoked A−β+ KPD. Although the first patient had concomitant HHS, DKA is not a common presentation in T2DM. Both patients were managed with standard DKA treatment. Their β-cell autoantibodies were negative, and C-peptides were normal after discharge, indicating the recovery of β-cell function. Their HbA1C dramatically improved after only three months of insulin therapy, and they were able to maintain remission for more than one year with diet and metformin.

## Conclusions

It is vital to recognize ketosis-prone diabetes clinically as many patients present with diabetic ketoacidosis, mainly if they are non-Caucasians. However, KPD is challenging to diagnose in the acute hospital setting. For patients with suspected ketosis-prone diabetes, β-cell autoantibodies and C-peptide level should be obtained from one to three weeks at the first outpatient visit to help determine KPD subgroups and predict patients’ prognoses. These patients should also follow up with an endocrinologist closely. This acute dysfunction is temporary in significant amounts of people and is followed by robust recovery of β-cell function. However, the mechanism of ketosis-prone diabetes remains unknown and merits further study.
